# Epidemiology of Rotavirus and Its Association with Other Etiological Agents of Enteric Infections in the Mexican Child Population (0–5 Years)

**DOI:** 10.3390/microorganisms14010172

**Published:** 2026-01-13

**Authors:** Larissa Fernandes-Matano, Luis Antonio Uribe-Noguez, Julio Elias Alvarado-Yaah, Angel Gustavo Salas-Lais, Clara Esperanza Santacruz-Tinoco, José Esteban Muñoz-Medina, Andrea Santos Coy-Arechavaleta

**Affiliations:** 1 División de Laboratorios Especializados, Instituto Mexicano del Seguro Social, Mexico City 07760, Mexico; larissamatano@gmail.com (L.F.-M.);; 2 Laboratorio Central de Epidemiología, Instituto Mexicano del Seguro Social, Mexico City 02990, Mexico

**Keywords:** gastrointestinal infection, rotavirus, watery diarrhea, dehydration, coinfections

## Abstract

Gastrointestinal infections are a major cause of morbidity worldwide. Rotavirus (RVA) is the most frequent cause of severe diarrheal disease in children and is associated with high direct and indirect costs. Symptoms of RVA infection are nonspecific, so diagnostic confirmation requires laboratory testing, which is not routinely performed due to its high cost. For this reason, only a small proportion of hospitalizations are correctly classified. In this context, this study was conducted to determine the prevalence of RVA and 19 other potential etiological agents in 642 samples from pediatric patients with gastrointestinal symptoms at the Social Security Mexican Institute (IMSS). The findings revealed a prevalence of RVA of 26.8%. When analyzing the 321 samples that were processed for the full panel, the positivity rate was 94.4% (for any of the etiological agents tested) and a high percentage of coinfections were detected (69.8%), including up to seven different etiological agents in the same child. The RVA was more frequent in children under 1 year of age, with higher circulation in winter and spring, while bacterial infections showed a seasonal trend in summer. The proportion of hospitalizations was higher in coinfections than in monoinfections, and RVA was the pathogen with the highest percentage of hospitalizations. The results emphasize the etiological complexity of gastrointestinal infections in the pediatric population, highlighting the importance of using multiplex diagnostic tests for appropriate clinical care and effective epidemiological control strategies.

## 1. Introduction

Gastrointestinal infections can cause diarrhea, a condition that is the third leading cause of death in children under five years old worldwide and confers significant morbidity, in addition to long-term adverse health effects [1]. Rotavirus (RVA) is the main etiological agent of severe diarrhea and gastroenteritis hospitalizations in children [2], which is associated with high direct and indirect costs, representing a major public health problem, particularly in winter and early spring [3].

The symptoms of rotavirus infection are usually nonspecific, such as diarrhea, vomiting, and fever, and the severity of the clinical presentation can vary considerably among infected children [4,5]. Therefore, definitive diagnosis requires laboratory testing, commonly enzyme immunoassays or latex agglutination tests, as well as PCR in some cases. However, these tests are not usually performed because a specific diagnosis is expensive and is believed to have no impact on treatment due to the lack of available antivirals. Consequently, the infection is managed through supportive therapies, such as fluid and electrolyte replacement [5,6].

Rotavirus vaccination was introduced worldwide and Mexico in 2006 [7]. During the pre-vaccination era, it was estimated that rotavirus caused approximately 75,000 hospitalizations and 2 million clinic visits per year [8]. Following the widespread use of vaccines, along with other improvements in treatment, nutrition, water quality, and sanitation, there was a reduction of up to 49.7% in diarrhea mortality between 1980 and 2015 in low- and middle-income countries [9]. However, even with a vaccine available, in 2016, rotavirus was responsible for more than 258 million episodes of diarrhea, with an incidence of 0.42 cases per child per year and caused approximately 128,500 deaths worldwide [8].

Nevertheless, other causes of diarrhea with vaccines in development, such as *Shigella*, enterotoxigenic *Escherichia coli* (ETEC), and norovirus, also cause a high proportion of pediatric diarrhea [10]. Furthermore, we cannot ignore the fact that vaccines are not always fully effective, as there is also circulation of heterotypic strains and a high number of mutations and genetic rearrangements of homotypic rotaviruses [11].

Due to the significant burden that gastrointestinal infections in children place on public health, especially those caused by rotavirus, and even though only a small proportion of hospitalizations are correctly classified [12,13], this study analyzed the prevalence of rotavirus, as well as 19 other important gastrointestinal pathogens, to update existing epidemiological data, using molecular and serological (antigen) tests.

## 2. Materials and Methods

### 2.1. Study Design

In this study, we analyzed the results of fecal samples from patients aged 0–5 years with gastrointestinal infection and suspected rotavirus infection, which were processed at the Central Epidemiology Laboratory (LCE) of the Social Security Mexican Institute (IMSS), and the data were deposited in the platforms of the Epidemiological Control System for Laboratories (SISCEP).

We analyzed 642 molecular and serological (antigen) tests for the detection of RVA, and 321 of these samples, selected randomly, were also processed for molecular detection of 19 other etiological agents (4 viruses, 11 bacteria, and 4 protozoans). The samples were received between June 2023 and October 2025. The criteria for sample acceptance could include any of the following operational definitions: Acute diarrheal disease (ADD): patient who demands medical attention for presenting 5 or more diarrheal stools in 24 h, for no more than 5 days with or without evidence of dehydration; Moderate ADD: patient who demands medical attention for presenting 5 or more diarrheal stools in 24 h, for no more than 5 days and who presents evidence of moderate dehydration; Severe ADD: patient who demands medical attention for showing 5 or more diarrheal stools in 24 h, for no more than 5 days and who has two or more of the following symptoms, vomiting (more than 5 in 24 h), dysenteric symptoms, temperature greater than 38 °C and evidence of moderate to severe dehydration [14].

**Figure 1:**
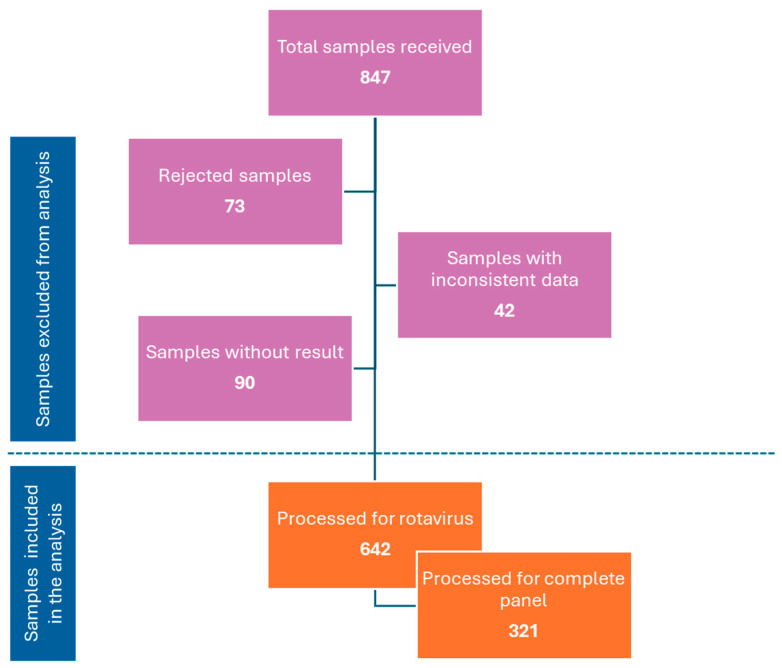
Database curation diagram. Summary of the samples excluded and included in the study. Complete panel = Rotavirus A (RVA), *Clostridium difficile* (CD), *Human mastadenovirus* (HAdV) (F40/41), *Mamastrovirus hominis* (MAstV), *Norovirus norwalkense* (NVN), *Sapovirus sapporoense* (SaV) (I, II, IV and V), *Campylobacter* spp. (CB), Enteroaggregative *Escherichia coli* (EAEC), Enteropathogenic *Escherichia coli* (EPEC), Enterotoxigenic *Escherichia coli lt/st* (ETEC), Shiga-like toxin-producing *Escherichia coli stx1/stx2* (STEC) (including *E. coli* O157, *Shigella*/Enteroinvasive *Escherichia coli* (EIEC), *Cryptosporidium* spp. (CP), *Yersinia enterocolitica* (YE), *Salmonella* sp. (SL), *Vibrio* spp. (VB) (including *Vibrio cholerae*), *Cyclospora cayetanensis* (CC), *Giardia lamblia* (GD), *Plesiomonas shigelloides* (PS) y *Entamoeba histolytica* (EH).

Figure 1 shows how the database was curated, removing inconsistent data and samples with any rejection reason applied by the laboratory, as well as the total number of samples analyzed for both RVA and the complete gastrointestinal panel.

The analysis of the information from the aforementioned platform excluded any data that could be traced to individuals, including the authors themselves.

### 2.2. Identification of Positive Cases

▪ *PREMIER^®^ ROTACLON^®^ (Cat. Number 696004) (Meridian Bioscience, Cincinnati, OH, USA) for the detection of rotavirus:* This test is an enzyme immunoassay used for the detection of rotavirus in stool samples. In this kit, the wells are coated with a monoclonal antibody directed against the antigen specific to this virus. The results were assigned by spectrophotometric determination. Samples with A450 absorbance units greater than 0.150 were considered positive. Samples with absorbances equal to or less than 0.150 were considered negative. The specificity and sensitivity of this kit are 92% and 100%, respectively, when compared with the results obtained by electron microscopy.
▪ *BioFire^®^ Gastrointestinal (GI) Panel Testing (Cat. Number 42489) (bioMérieux, Marcy l’Étoile, France):* This kit was used for the detection of 20 pathogens associated with gastroenteritis. Nucleic acids are extracted from stool samples using mechanical and chemical lysis, followed by purification using standard magnetic bead technology. Then, nested multiplex RT-PCR followed by high resolution melting analysis was used to confirm the identity of the amplified product. The software version 3.2.4.0 of the BIOFIRE^®^ FILMARRAY^®^ 2.0 (bioMerieux, Marcy-l'Étoile, France) automatically interprets the results. . The observed sensitivity and specificity of the melt detector are greater than 98.92% and 99.99%, respectively. For the Analysis Software (v. 3.2.4.0), 99.34% and 99.99%, respectively.

### 2.3. Statistical Analysis

Descriptive statistics were used to report frequencies and percentages; these were calculated with their respective 95% confidence intervals. Chi-square tests were used to compare categorical variables. *p*-values < 0.05 were considered significant in all cases. IBM SPSS Statistics 24.0, RStudio (version 2023.06.1+524, Boston, MA, USA), and Microsoft Excel (version 16.66.1, Redmond, WA, USA) were used for the analysis and generation of graphs.

## 3. Results

### 3.1. Identification of Positive Cases

The demographic data summary is shown in Table 1. Of the 642 samples analyzed for rotavirus, 57.3% (368/642) belonged to male patients, indicating that boys sought medical attention more frequently due to gastrointestinal infections. Although not statistically significant, overall and rotavirus positivity rates were higher in girls (*p* > 0.05).

Patients were divided into age groups according to their age at the time of sample collection (≤11 months; ≥12 and <23 months; ≥24 and ≤60 months), with only children aged 0 to 5 years included. The younger the patient, the higher the suspicion of rotavirus infection, as the number of processed samples decreased with age. However, the differences in rotavirus prevalence were not significant between age groups (*p* > 0.05). On the other hand, the prevalence of other etiological agents was statistically lower in children ≤ 11 months (*p* < 0.05). The prevalence of coinfections in the analysis performed on the samples processed for the complete panel was 69.8% (224/321) and involved all the agents.

Data on the clinical status of the patients included in the study are also shown in Table 1, 642 samples were processed for RVA; of these samples, 321 were also processed for the full panel, most of the 642 patients were hospitalized 64.8% (416/642), outpatient cases were being the least frequent 35.2% (226/642), and no deaths.

### 3.2. Confirmation of Rotavirus and Other Infectious Agents and Their Seasonality

As shown in Table 1, all samples included in the study were processed for RVA detection, yielding an overall prevalence of 26.8% (172/642). However, the full panel was only processed in 321 samples, resulting in an overall prevalence of 94.4% (303/321) of all the pathogens included in the panel. All the agents identified and their prevalences are shown in Table 2.

In RVA-positive cases, the number of hospitalizations was 2.7 times higher than in outpatient cases. In positive patients for pathogens other than RVA, hospitalizations were 2.04 times higher than in outpatient cases (*p* < 0.05), indicating that RVA infections typically require more hospitalizations than infections with other pathogens. However, when analyzing the hospitalization rate for each pathogen individually, 11 of them had hospitalization rates exceeding 70%.

On the other hand, when comparing the hospitalization rate for monoinfections to that for coinfections, we found (51/79) 64.6% and (166/224) 74.1%, respectively, which may indicate that coinfections can have significant clinical implications.

### 3.3. Seasonality of Detected Pathogens

**Figure 2:**
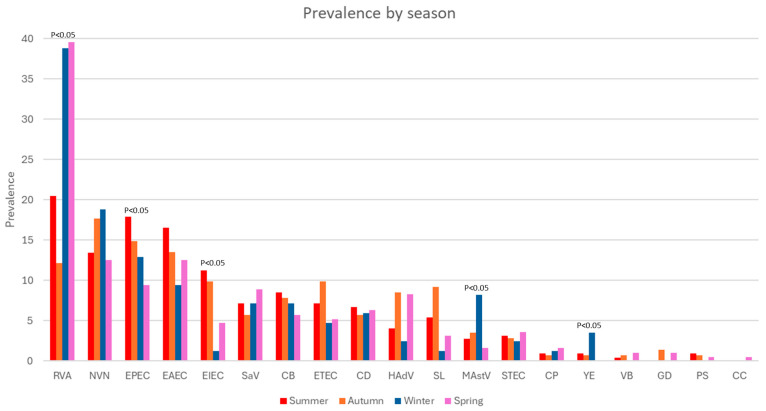
Prevalence of the etiological agents identified in the analyzed cases of gastrointestinal infection, according to the season.

The prevalence of RVA differed statistically depending on the season. In this study, we identified winter and spring as the seasons with the highest proportion of RVA-positive cases 38.6% (124/321) and 39.6% (127/321), respectively (Figure 2). We also identified seasonal differences in prevalence for only four other agents: EPEC, with the highest prevalence in summer and the lowest in autumn; EIEC, with the highest prevalence in summer and the lowest in winter; MAstV, with the highest seroprevalence in winter and the lowest in autumn; and YE, with the highest prevalence in winter and no detections in autumn. Analyzing only the samples processed for the complete panel, we generally found that bacterial infections had a higher prevalence in summer 86.6% (278/321); *p* < 0.05, while viruses had a higher prevalence in winter 89.4% (287/321); *p* < 0.05 (Table 3).

### 3.4. Coinfections

**Figure 3:**
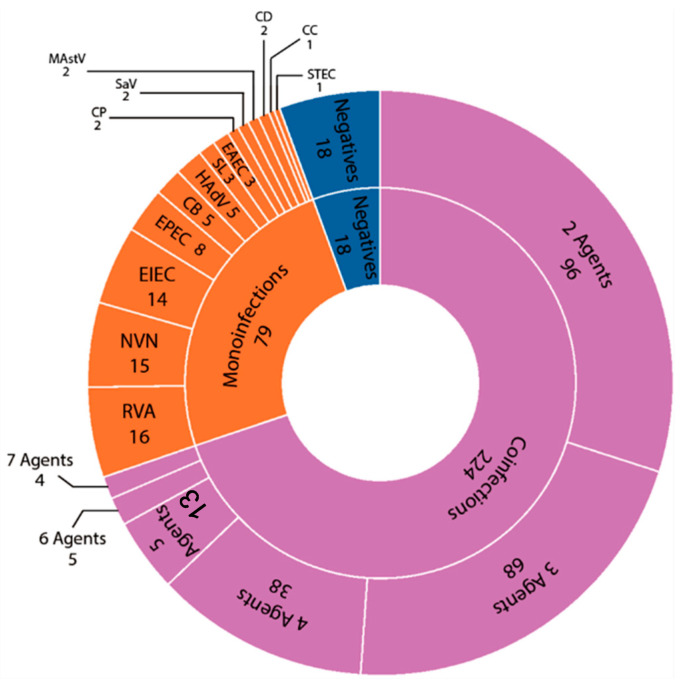
Outcome and type of infections detected in the samples processed for the complete panel (n = 321).

Figure 3 summarizes the number of detections in the 321 samples processed for the full panel. Negative cases were the minority, only 5.6% (18/321). In the 24.6% (79/321) of monoinfections detected in the sample pool processed for the full panel, 14 different agents were identified, the most prevalent being RVA 5.0% (16/321), NVN 4.7% (15/321), and EIEC 4.4% (14/321). The agents identified only in coinfection cases were: ETEC, YE, VB, GD, and PS.

As shown in Table 1, cases of coinfection with 2 to 7 agents were identified in the same child. Coinfections represented most cases analyzed for the entire panel 69.8% (224/321) and 73.9% (224/303) of the positive samples.

Although 18 of the analyzed agents were present in coinfections, the percentages of participation were significantly different (*p* < 0.05). A high percentage of the positivity among the etiological agents was attributed to coinfection cases (Table 4).

**Figure 4:**
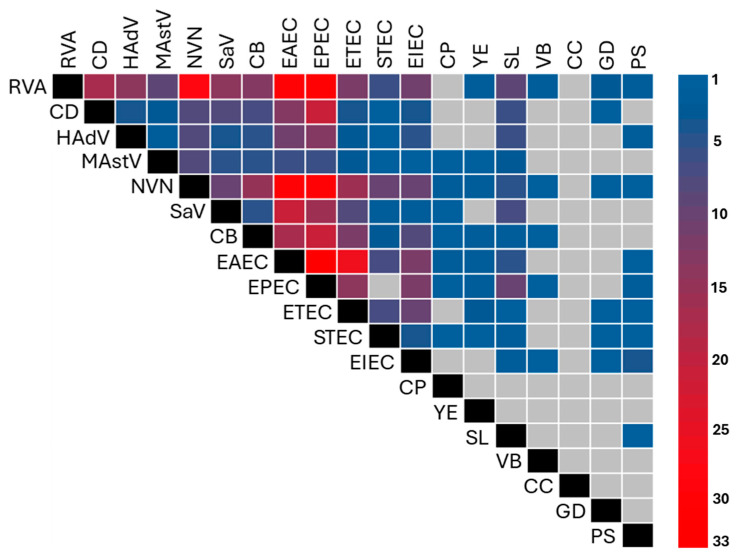
Heat map of the frequency of combinations between etiological agents of gastrointestinal infections. The color scale refers to the number of times the combination of pathogens occurred; color gray indicates no coinfections.

Regarding the possible combinations of agents, some appear to be more frequent than others, such as EAEC + EPEC, RVA + EPEC, NVN + ETEC, NVN + EPEC, and RVA + EAEC, as shown in Figure 4.

## 4. Discussion

In this study, we analyzed the prevalence of RVA and 19 other pathogens in samples from pediatric patients (up to 5 years old) who presented to the Social Security Mexican Institute (IMSS) with acute diarrheal disease, moderate acute diarrheal disease, and severe acute diarrheal disease.

Before and during this study, it was difficult to find up-to-date information on the prevalence of RVA, and moreover, on the etiology of gastrointestinal infections.

Although the introduction of the RVA vaccine has substantially reduced the global burden of disease caused by this virus, the efficacy observed in low- and middle-income countries is often lower than in developed countries [12,13,15,16]. A 2021 systematic review of the evidence from 15 years of rotavirus vaccination in Mexico showed that the vaccines are safe, immunogenic, and effective, preventing severe cases in 80% to 100% of cases and reducing the need for hospitalization by 42% [15]. A key factor for the success of vaccination programs is adherence to the schedules outlined in the National Immunization Program (NIP). In Mexico, two rotavirus vaccines have been used in the NIP: Rotarix (two doses, 2 and 4 months of age) from 2006 to 2011, and RotaTeq (three doses, 2, 4, and 6 months of age) since 2011 [17,18]. In a study conducted to evaluate the coverage of these two vaccines, it was observed that in 2010, 80.7% received at least one dose of Rotarix, 75.6% received both doses, and 57.0% received both doses according to the schedule. In 2012, 85.7% received at least one dose of RotaTeq, 61.0% received all three doses, and 43.2% received all three doses according to the schedule [19]. During the preparation of this work, we did not have vaccination coverage data for the patients included in this study, nor recent information on the subject in the Mexican population. Therefore, our observations cannot be directly linked to the impact of the vaccine; however, the findings emphasize the need to strengthen vaccination campaigns and ensure compliance with the complete vaccination schedule, especially in vulnerable populations, as well as the need for studies that evaluate the effectiveness of the vaccine in Mexican children.

Our findings show that, although RVA was the main agent detected (26.8% of all samples analyzed and 34.9% of samples processed by the complete molecular panel), in most cases, the clinical picture cannot be attributed solely to this virus, because we frequently detected combinations of viruses, bacteria, and protozoa (69.8% of samples processed by the complete molecular panel and 73.9% of positive samples). Furthermore, the percentage of hospitalizations was higher in coinfections than in monoinfections (2.7 times higher in the case of RVA and 2.04 times higher for the other identified agents), which calls into question the current clinical practice of ordering diagnostic studies almost exclusively for RVA [20].

In other countries, RVA continues to be the main etiological agent of diarrhea identified in children, despite the availability of vaccination [21,22]. A 2019 study in India found that after RVA, the main etiological agents were, HAdV, EIEC, NVN, SAV, and CP were also detected [23]. This research group also detected coinfections of RVA with bacteria, which were associated with a longer duration of illness, and coinfections involving protozoa were associated with a higher hospitalization rate [21]. In Africa, RVA is also identified as the main etiological agent of gastric infections in children, followed by EIEC, NVN, CB, HAdV, ETEC, among others, as well as a high rate of coinfections [22].

Regarding the seasonality of the different etiological agents tested, there was already evidence of an increase in cases caused by RVA in winter. In fact, we confirmed that winter and spring are the times of year with the highest circulation of this virus and, in general, of the other viruses detected, while during the summer, we observed a higher frequency of bacterial pathogens [24]. The results obtained in a study conducted by Angeles G et al., at InDRE in 2002, also show that the most frequent *E. coli* pathotypes in Mexico are detected more frequently during warm and humid seasons [25]. These findings reinforce the epidemiological validity of our results and confirm the seasonal differences between bacteria and viruses. Therefore, our results can serve as a preliminary guide for etiological diagnosis and could be used as a tool to strengthen clinical algorithms, especially in areas where diagnostic resources are limited.

Conducting this type of study more frequently and on a larger scale, both in Mexico and around the world, would undoubtedly have important implications for epidemiological surveillance, clinical decision-making, and the implementation of health policies, particularly in countries where the morbidity of gastrointestinal infections is high and there is significant underreporting of these infections.

In Mexico, for example, the lack of recent studies on the etiology and prevalence of various enteric pathogens circulating in the country contributes to a lack of reliable information for estimating the contribution of each type of microorganism (viruses, bacteria, and protozoa) to the morbidity and burden of gastrointestinal infections. In fact, it has been documented that only about 47% of hospitalized cases caused by RVA are correctly classified, not to mention the importance of asymptomatic infections for transmission [24,26]. Furthermore, laboratory diagnostic confirmation is infrequent due to economic limitations and the widespread perception that treatment will not significantly alter the course of intestinal infections. In addition, in Mexico, it is quite common to use only diagnostic techniques such as PAGE or EIA, which have lower sensitivity compared to molecular techniques, causing false negatives and subsequent underreporting [27]. This reinforces the need to adopt more sensitive and standardized diagnostic methodologies to improve national surveillance.

The existing bias in current epidemiological data on intestinal infections is exacerbated by the guidelines for diagnostic confirmation [14,28], since rotavirus infection shares the operational case definition with all acute diarrheal diseases. That is, even though the symptoms are similar in infections caused by different pathogens, the laboratory only performs the test for rotavirus. This, coupled with the fact that only 10% of negative samples undergo differential diagnosis for other intestinal viruses and only another 20% are tested for parasites, means that no percentage is allocated for the detection of bacterial pathogens. This practice limits the detection of coinfections and their clinical significance, preventing patients from receiving more appropriate treatment, increasing the likelihood of prolonged hospital stays, raising healthcare costs, and increasing the probability of death. Therefore, the evidence presented underscores the urgent need to broaden the panel of pathogens investigated in pediatric patients with diarrhea, especially when significant clinical manifestations are present, and hospitalization is required.

## 5. Conclusions

Taken together, our results demonstrate the importance of implementing more sensitive diagnostic tests and expanded screening panels for enteric pathogens, as they reveal a very high percentage of coinfections involving various pathogens that cause diarrhea in children under 5 years of age. Furthermore, we demonstrate that considering seasonal patterns to guide diagnostic testing can be useful when resources are limited. The identification of rotavirus (RVA) as the main etiological agent, even with a vaccine available for its prevention, underscores the need to strengthen vaccination efforts. All these actions would contribute to more precise clinical management, a reduction in complications, and a decrease in the costs associated with hospitalization in children under five years of age in Mexico. In addition, improving epidemiological surveillance to report more accurate data on the etiology of these types of infections would help understand the clinical significance of the many coinfections detected in this study, as well as aid in the design of alternative diagnostic tests.

## Figures and Tables

**Table 1:** Summary of demographic and clinical status data.

	ROTAVIRUS		COMPLETE PANEL	
	PROCESSED (All Samples Processed for Rotavirus)	POSITIVITY (for Rotavirus Only)	PROCESSED (Only Samples Processed for the Complete Panel)	POSITIVITY (for Any of the Tested Agents)
TOTAL	642 (100%)	172 (26.8%)	321 (100%)	303 (94.4%)
Women	274 (42.7%)	75 (27.4%)	134 (41.7%)	128 (95.5%)
Man	368 (57.3%)	97 (26.4%)	187 (58.3%)	175 (93.6%)
AGE GROUP				
≤11 months	237 (36.9%)	74 (31.2%)	127 (39.6%)	114 (89.8%)
≥12 and <23 months	225 (35.0%)	54 (24.0%)	114 (35.5%)	112 (98.2%)
≥24 and ≤60 months	180 (28.0%)	44 (24.4%)	80 (24.9%)	77 (96.3%)
REGION				
Central	225 (35.0%)	53 (23.6%)	109 (34.0%)	102 (93.6%)
Northeast	78 (12.2%)	21 (26.9%)	39 (12.1%)	39 (100.0%)
Northwest	98 (15.3%)	25 (25.5%)	52 (16.2%)	50 (96.1%)
West	121 (18.8%)	38 (31.4%)	66 (20.6%)	63 (95.4%)
Southeast	120 (18.7%)	35 (29.2%)	55 (17.1%)	49 (89.1%)
CLINICAL STATUS				
Outpatient	226 (35.2%)	47 (20.8%)	93 (29.0%)	86 (92.4%)
Hospitalized	416 (64.8%)	125 (30.0%)	228 (71.0%)	217 (95.2%)
RESULTS				
Negatives	470 (73.2%)		18 (5.6%)	
Positives	172 (26.8%)		303 (94.4%)	
Positives to 1 agent	76 (11.8% of the total samples processed and 44.2% of the positive samples)		79 (24.6% of the total samples processed and 26.1% of the positive samples)	
Coinfections	96 (14.9% of the total samples processed and 55.8% of the positive samples)		224 (69.8% of the total samples processed and 73.9% of the positive samples)	

**Table 2:** Analysis of the prevalence and hospitalization rate for each pathogen.

Etiological Agent	Agent Type	N	Prevalence (%)	CI (%)	Hospitalizations (N)	Percentage (%)	CI (%)
RVA	Virus	172 (of 642)	26.8%	23.4–30.2	125	72.7%	66.0–79.4
		112 (of 321)	34.9%	29.7–40.1	90	80.3%	72.9–87.7
NVN	Virus	95	29.6%	24.6–34.6	71	74.7%	66.0–83.4
EPEC	Bacteria	90	28.0%	23.1–33.0	68	75.6%	66.7–84.5
EAEC	Bacteria	88	27.4%	22.5–32.3	54	61.4%	51.2–71.6
EIEC	Bacteria	49	15.3%	11.3–19.2	35	71.4%	58.7–84.0
SAV	Virus	47	14.6%	10.8–18.5	29	61.7%	47.8–75.6
CB	Bacteria	47	14.6%	10.8–18.5	32	68.1%	54.8–81.4
ETEC	Bacteria	44	13.7%	9.9–17.5	33	75.0%	62.2–87.8
CD	Bacteria	40	12.5%	8.8–16.1	29	72.5%	58.7–86.3
HAdV	Virus	39	12.1%	8.6–15.7	29	74.4%	60.7–88.1
SL	Bacteria	32	10.0%	6.7–13.2	24	75.0%	60.0–90.0
MAstV	Virus	21	6.5%	3.8–9.2	16	76.2%	58.0–94.4
STEC	Bacteria	20	6.2%	3.6–8.9	15	75.0%	56.0–94.0
CP	Protozoan	7	2.2%	0.6–3.8	3	42.9%	21.2–64.6
YE	Bacteria	6	1.9%	0.4–3.4	5	83.3%	53.5–100
VB	Bacteria	4	1.2%	0.0–2.5	2	50.0%	1.0–99.0
GD	Protozoan	4	1.2%	0.0–2.5	1	25.0%	0.0–67.4
PS	Bacteria	4	1.2%	0.0–2.5	3	75.0%	32.6–100
CC	Protozoan	1	0.3%	−0.3–0.9	1	100%	NA
EH	Protozoan	0	0%	NA	0	0%	NA

NA: Not applicable due to insufficient sample size. N: Number of samples.

**Table 3:** Analysis of prevalence according to seasonality, according to the type of microorganism.

	Prevalence (%)		
	Virus	Bacteria	Protozoan
Summer	62.3	86.6	1.9
Autumn	57.5	76.3	3.8
Winter	89.4	66.7	2.6
Spring	83.3	59.4	6.2

**Table 4:** Prevalence and coinfection data for the agents analyzed for the complete panel.

Etiological Agent	Total	Positives	Coinfections	Percentage of Their Positivity Attributed to Coinfections	Percentage of Total Attributed to Coinfections
RVA	321	112	96	85.70%	29.90%
NVN	321	95	80	84.20%	24.90%
EPEC	321	90	82	91.10%	25.50%
EAEC	321	88	85	96.60%	26.50%
EIEC	321	49	35	71.40%	10.90%
SaV	321	47	45	95.70%	14.00%
CB	321	47	42	89.40%	13.10%
ETEC	321	44	44	100%	13.70%
CD	321	40	38	95.00%	11.80%
HAdV	321	39	34	87.20%	10.60%
SL	321	32	29	90.60%	9.00%
MAstV	321	21	19	90.50%	5.90%
STEC	321	20	19	95.00%	5.90%
CP	321	7	5	71.40%	1.60%
YE	321	6	6	100%	1.90%
VB	321	4	4	100%	1.20%
GD	321	4	4	100%	1.20%
PS	321	4	4	100%	1.20%
CC	321	1	0	0%	0%

## Data Availability

All data necessary to replicate the analyses performed in this study are available at https://figshare.com/articles/dataset/_b_Rotavirus_and_its_association_with_other_etiological_agents_b_/30625283?file=59603531; https://doi.org/10.6084/m9.figshare.30625283, accessed on 6 January 2026.
